# Mechanism of Fcγ Receptor-Mediated Trogocytosis-Based False-Positive Results in Flow Cytometry

**DOI:** 10.1371/journal.pone.0052918

**Published:** 2012-12-27

**Authors:** Sakiko Masuda, Sari Iwasaki, Utano Tomaru, Juri Sato, Ai Kawakami, Kana Ichijo, Sayuri Sogo, Tomohisa Baba, Kazuaki Katsumata, Masanori Kasahara, Akihiro Ishizu

**Affiliations:** 1 Graduate School of Health Sciences, Hokkaido University, Kita-ku, Sapporo, Japan; 2 Faculty of Health Sciences, Hokkaido University, Kita-ku, Sapporo, Japan; 3 Department of Pathology, Hokkaido University Graduate School of Medicine, Kita-ku, Sapporo, Japan; 4 School of Health Sciences, Hokkaido University, Kita-ku, Sapporo, Japan; National Institute of Infectious Diseases, Japan

## Abstract

The whole blood erythrocyte lysis method is the most common protocol of sample preparation for flow cytometry (FCM). Although this method has many virtues, our recent study has demonstrated false-positive results when surface markers of monocytes were examined by this method due to the phenomenon called Fcγ receptor (FcγR)-mediated trogocytosis. In the present study, similar FcγR-mediated trogocytosis-based false-positive results have been demonstrated when granulocytes were focused on instead of monocytes. These findings indicated that not only monocytes but also granulocytes, the largest population with FcγR expression in peripheral blood, could perform FcγR-mediated trogocytosis. Since the capacity of FcγR-mediated trogocytosis was different among blood samples, identification of factors that could regulate the occurrence of FcγR-mediated trogocytosis should be important for the quality control of FCM. Our studies have suggested that such factors are present in the serum. In order to identify the serum factors, we employed the *in vitro* model of FcγR-mediated trogocytosis using granulocytes. Investigation with this model determined the serum factors as heat-labile molecules with molecular weight of more than 100 kDa. Complements in the classical pathway were initially assumed as candidates; however, the C1 inhibitor did not yield an obvious influence on FcγR-mediated trogocytosis. On the other hand, although immunoglobulin ought to be resistant to heat inactivation, the inhibitor of human anti-mouse antibodies (HAMA) effectively blocked FcγR-mediated trogocytosis. Moreover, the inhibition rates were significantly higher in HAMA^high^ serum than HAMA^low^ serum. The collective findings suggested the involvement of heterophilic antibodies such as HAMA in the mechanism of false-positive results in FCM due to FcγR-mediated trogocytosis.

## Introduction

Flow cytometry (FCM) is an indispensable analytical method to detect cell surface markers in the field of laboratory medicine. Identification of surface markers of peripheral blood leukocytes is important particularly in the diagnosis of hematological malignancy. Currently, whole blood erythrocyte lysis method is the most common protocol of sample preparation for FCM [1]. In this method, antibodies (Abs) for detection, usually mouse anti-human Abs, are first added to whole blood samples. Erythrocytes are next removed chemically, and then leukocytes are subjected to FCM. This method is quick and easy, utilizes a small sample volume, and does not change the leukocyte fraction. Another merit of this method is no need to use Fcγ receptor (FcγR) blockers because considerable amount of human IgG (8–16 mg/ml), which can mask FcγRs, is included in the sample itself. However, our recent study has pointed out that false-positive results occurred when monocytes were examined by this method [2].

In our earlier study, CD8^+^ monocytes were detected in human peripheral blood samples when FCM was performed using mouse anti-human CD8 and CD14 Abs by whole blood erythrocyte lysis method [2]. Since peripheral blood monocytes (CD14^+^ cells) did not produce CD8 mRNAs, and CD8 molecules were not detected on purified monocytes, the result was regarded as false-positive. Analysis of the mechanism revealed that CD8 molecules on T cells were transferred to monocytes when whole blood samples were incubated with the anti-CD8 Ab. For completion of the CD8 translocation from T cells to monocytes, cell-to-cell contact between T cells and monocytes was required. Moreover, since the CD8 translocation was not induced by F(ab′)_2_ of the anti-CD8 Ab and was inhibited by blocking Abs to FcγRII (CD32), the binding of the Fc portion of the Ab and FcγRII (CD32) on monocytes was also involved in the mechanism. In addition, CD3 and T cell receptor (TCR) molecules that constituted a functional complex with CD8 were transferred from T cells to monocytes accompanied by the translocation of CD8.

Leukocytes can “gnaw away” the plasma membrane of other cells [3]. This phenomenon, called trogocytosis, occurs subsequent to cell-to-cell adhesion. To date, two mechanisms of trogocytosis have been identified; one is adhesion molecule-mediated trogocytosis and the other is FcγR-mediated trogocytosis [4]. The former occurs following the adhesion of cell surface molecules with the specific ligands on other cells; whereas, the latter happens when Abs bridge the antigens (Ags) and FcγRs between the cells. The major difference of FcγR-mediated trogocytosis from adhesion molecule-mediated trogocytosis is the intervention of Abs. Accordingly, the false-positive result of CD8 detection on monocytes in the whole blood erythrocyte lysis method in FCM is considered to be caused by FcγR-mediated trogocytosis.

In the present study, we demonstrated that similar FcγR-mediated trogocytosis-based false-positive results occurred when granulocytes were focused on instead of monocytes, using anti-CD8 and anti-CD15 Abs. Although granulocytes (CD15^+^ polymorphonuclear cells (PMNs)) cannot express CD8, some granulocytes appeared to express CD8. Since granulocytes and T cells could be distinguished by different gates in FCM, contamination of T cells in the granulocyte population was not likely to occur. The present study demonstrated that the detection of CD8^+^ granulocytes resulted from FcγR-mediated trogocytosis; thus, this study indicated that not only monocytes but also granulocytes, the largest population with FcγR expression in peripheral blood, could perform FcγR-mediated trogocytosis.

The aim of this study is to determine the mechanism of FcγR-mediated trogocytosis. For that purpose, putative factors that critically contributed to FcγR-mediated trogocytosis were investigated by employing the *in vitro* CD8 translocation model from T cells to granulocytes (granulocyte FcγR-mediated trogocytosis model). As a result, serum heat-labile factors with molecular weight of more than 100 kDa were the proposed candidates that could contribute to FcγR-mediated trogocytosis. Among such factors, complements in the classical pathway were first nominated. Next, although immunoglobulin ought to be heat-resistant, heterophilic Abs, such as human anti-mouse Abs (HAMA), were examined based on the consideration that diverse factors could participate in FcγR-mediated trogocytosis.

## Results

### Detection of CD8^+^ granulocytes in human peripheral blood samples

**Figure 1:**
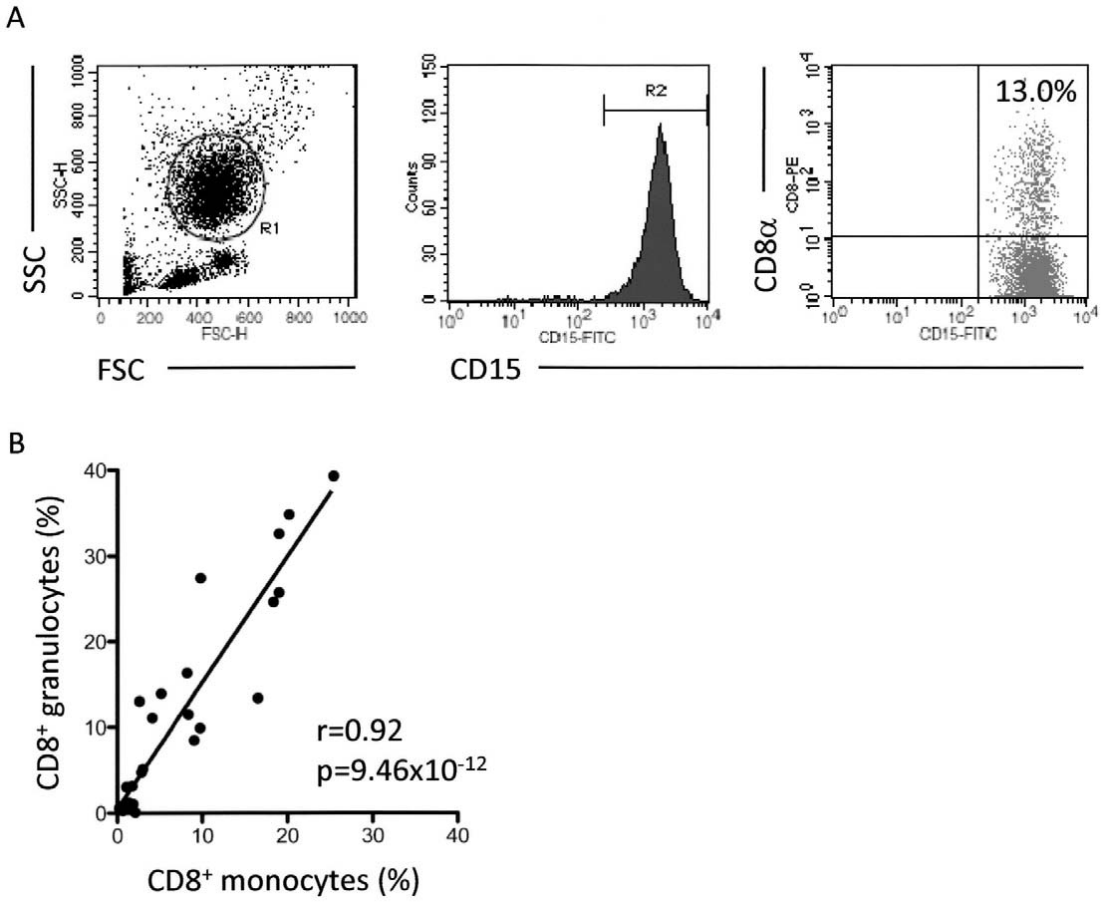
Detection of CD8^+^ granulocytes and correlation to CD8^+^ monocytes. (**A**) Detection of CD8^+^ granulocytes in human peripheral blood samples. Heparinized whole blood samples were made to react with the PE-labeled anti-CD8α Ab (HIT8a). After depletion of erythrocytes, the cells were re-suspended in PBS, and then allowed to react with the FITC-labeled anti-CD15 Ab (H198). PE-labeled mouse IgG1 and FITC-labeled mouse IgM were used as isotype-matched controls for HIT8a and H198, respectively. The cells gated in R1 were regarded as PMNs. Among the PMNs, granulocytes were characterized by the high level of expression of CD15 (gated in R2). The cells in both R1 and R2 gates were examined for the expression of CD8α. (**B**) Correlation of CD8^+^ granulocytes and CD8^+^ monocytes (n = 32, r = 0.92, p = 9.46×10^−12^ in Pearson correlation test).

Heparinized peripheral blood samples from healthy volunteers were made to react with the PE-labeled anti-CD8α Ab. After removal of erythrocytes, the cells were re-suspended in PBS, and then allowed to react with the FITC-labeled anti-CD15 Ab, followed by serving for FCM. The representative FACS profile is shown in Figure 1A. The cells gated in region 1 (R1) were regarded as single-cell PMNs. Among the PMNs, granulocytes were characterized by the high level of expression of CD15 (gated in region 2 (R2)). The cells in both R1 and R2 gates were examined for the expression of CD8α. In the present case, 13.0% of granulocytes showed positive staining for CD8α. CD8α^+^CD15^+^ PMNs designated as CD8^+^ granulocytes were detected in all samples examined (n = 32). CD8^+^ granulocytes were also detected when other Abs to CD8α or CD8β were used (Figure S1); therefore, this phenomenon was not limited to certain anti-CD8α Ab. The detection rates of CD8^+^ granulocytes in whole granulocytes (CD15^+^ PMNs) exhibited individual variations ranging from 1% to 40% (mean ± SD: 10.2±11.8%). Interestingly, the rate of CD8^+^ granulocytes in individuals was well correlated to that of CD8^+^ monocytes (Figure 1B, r = 0.92, p = 9.46×10^−12^ in Pearson correlation test); therefore, it is considered that an identical mechanism could be implicated in the detection of CD8^+^ granulocytes and CD8^+^ monocytes.

### Detection of CD8^+^ granulocytes as model of FcγR-mediated trogocytosis

**Figure 2:**
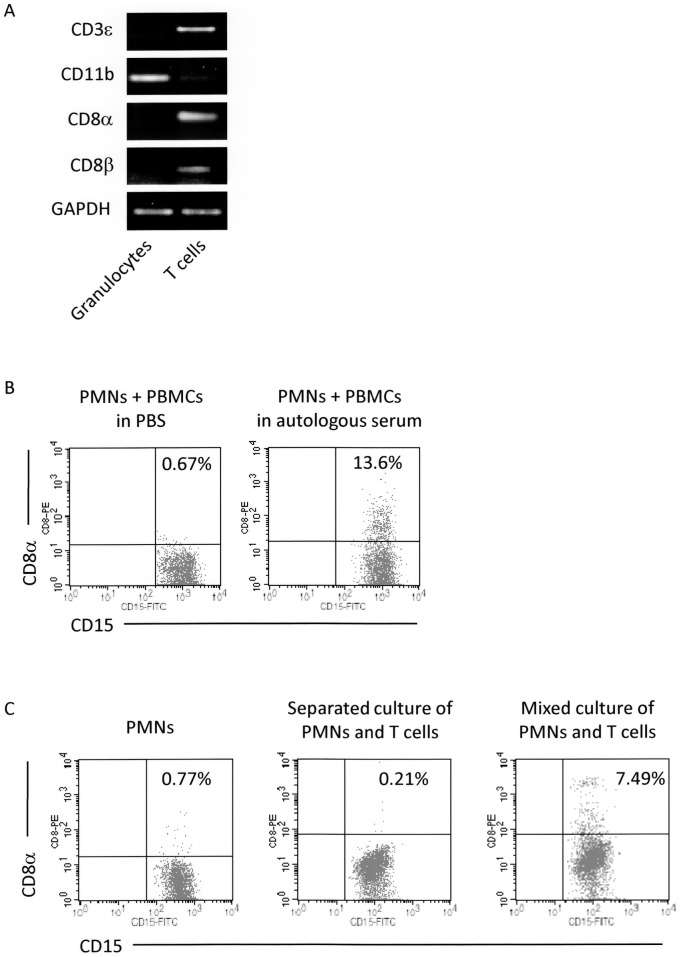
Requirement of serum and cell-to-cell interaction with T cells in detection of CD8^+^ granulocytes. (**A**) mRNA expressions of CD3ε, CD11b, CD8α, and CD8β in granulocytes (CD15^+^ PMNs) and T cells (CD3^+^ cells) determined by RT-PCR. These cells were separated from blood samples as described in Materials and Methods. The quality of RNA samples was verified by the expression of GAPDH. (**B**) Requirement of serum for detection of CD8^+^ granulocytes. PMNs and PBMCs separated from heparinized peripheral blood were mixed in PBS or autologous serum. These cells were incubated with the PE-labeled anti-CD8α Ab (HIT8a). After removal of unbound Abs, the cells were re-suspended in PBS followed by reaction with the FITC-labeled anti-CD15 Ab (H198), and then served for FCM. (**C**) Requirement of cell-to-cell contact with T cells for detection of CD8^+^ granulocytes. PMNs and T cells were separated from heparinized blood samples. PMNs were cultured with or without T cells in the autologous serum. In the co-culture wells, PMNs were cultured separately from T cells using the transwell chambers or mixed together with T cells. Subsequently, the cells were made to react with the PE-labeled anti-CD8α Ab (HIT8a). After removal of unbound Abs, the cells were re-suspended in PBS followed by reaction with the FITC-labeled anti-CD15 Ab (H198), and then served for FCM. These experiments were repeated 3 times. PE-labeled mouse IgG1 and FITC-labeled mouse IgM were used as isotype-matched controls for HIT8a and H198, respectively.

The majority of CD8 molecules are heterodimers composed of CD8α and CD8β. To confirm that CD8 molecules detected on granulocytes were not produced by the cells, the mRNA expression of CD8α and CD8β was examined in CD15^+^ PMNs containing CD8^+^ granulocytes (Figure S2). CD3^+^ cells containing CD8^+^ T cells were also obtained as positive controls. The RT-PCR showed that mRNA of neither CD8α nor CD8β was expressed in CD15^+^ PMNs containing CD8^+^ granulocytes; thus, this suggested that CD8 molecules were not produced by granulocytes (Figure 2A).

Requirement of serum for detection of CD8^+^ granulocytes is shown in Figure 2B. CD8^+^ granulocytes were hardly detected when the anti-CD8α Ab was added to leukocytes (PMNs+peripheral blood mononuclear cells (PBMCs)), which had been separated from whole blood samples and re-suspended in PBS (0.67% in the present case). On the contrary, CD8^+^ granulocytes were detected when the anti-CD8α Ab was added to leukocytes (PMNs+PBMCs) re-suspended in the autologous serum (13.6% in the present case).

These findings suggested that CD8 molecules expressed on cells other than granulocytes were transferred to granulocytes under the presence of serum and anti-CD8α Ab. Majority of CD8 molecules are expressed on T cells. To demonstrate that CD8 molecules detected on granulocytes are derived from T cells and that cell-to-cell contact between granulocytes and T cells is required for the detection of CD8^+^ granulocytes, PMNs and T cells were separated from whole blood samples. Subsequently, PMNs were cultured with or without T cells under the presence of serum and anti-CD8α Ab. In the co-culture wells, PMNs were incubated separately from T cells using transwell chambers or mixed together with T cells. The representative results are shown in Figure 2C. In the present case, few granulocytes showed positive staining for CD8α when PMNs were cultured alone or when PMNs were incubated separately from T cells (0.77% and 0.21%, respectively). On the other hand, the rate of CD8^+^ granulocytes was 7.49% when PMNs and T cells were mixed together. As shown in Figure S3, only PMNs with characteristic single-cell FSC/SSC profile were included in the assay to avoid contamination of T cell-granulocyte doublets. These findings indicated that CD8 molecules detected on granulocytes were derived from T cells, and that cell-to-cell contact between granulocytes and T cells was required for the detection of CD8^+^ granulocytes.

**Figure 3:**
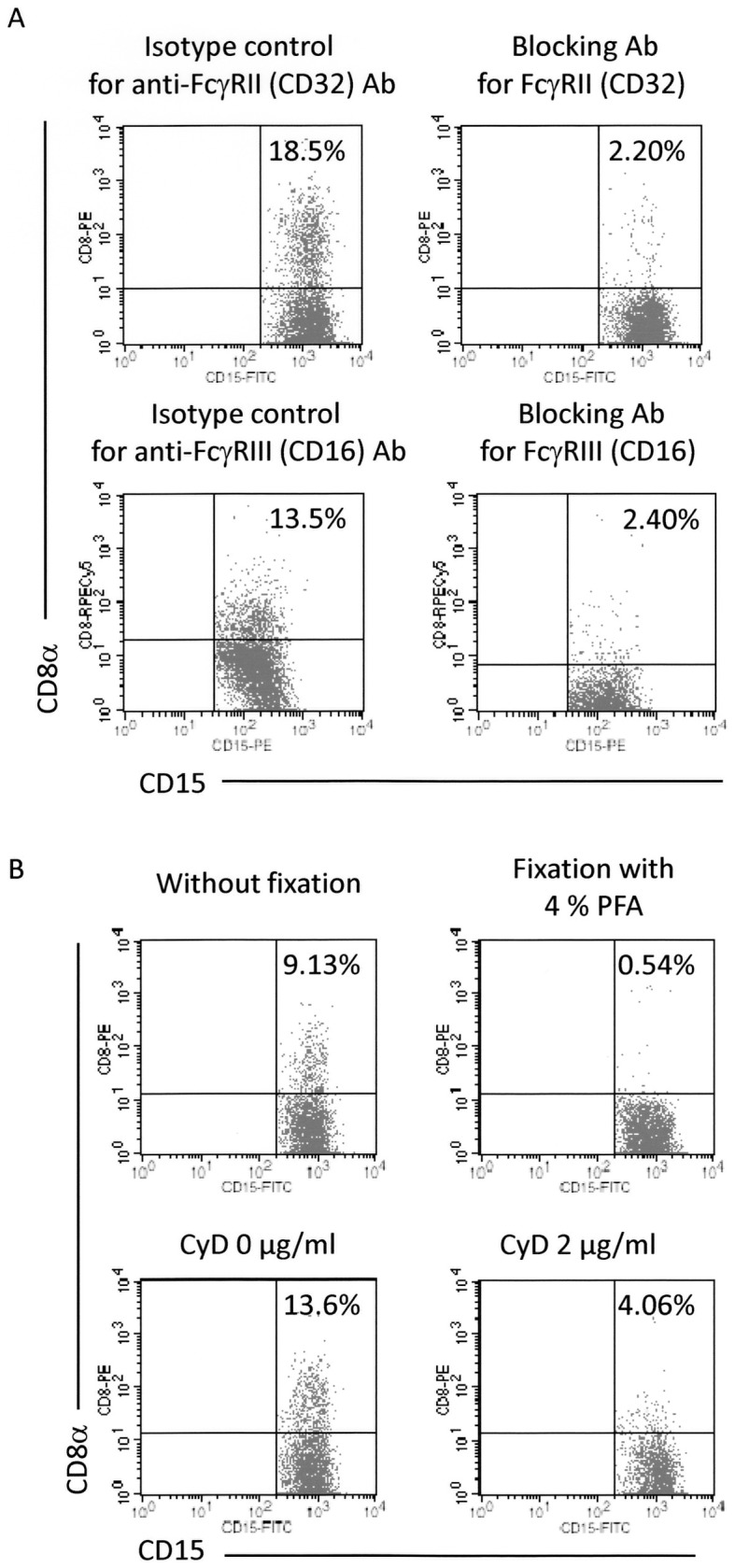
Requirement of FcγRs, dynamism of plasma membrane, and actin recruitment in detection of CD8^+^ granulocytes. (**A**) Involvement of FcγRII (CD32) and FcγRIII (CD16) in the detection of CD8^+^ granulocytes. Heparinized blood samples were pre-incubated with the anti- FcγRII (CD32) Ab (AT10) or with the anti-FcγRIII (CD16) Ab (3G8). After the pre-incubation, the samples were made to react with the PE or PECy5-labeled anti-CD8α Ab (HIT8a). After depletion of erythrocytes, the cells were re-suspended in PBS followed by reaction with the FITC or PE-labeled anti-CD15 Ab (H198), and then served for FCM. PE or PECy5-labeled mouse IgG1 and FITC or PE-labeled mouse IgM were used as isotype-matched controls for HIT8a and H198, respectively. (**B**) Effects of plasma membrane fixation and inhibition of actin recruitment in the detection of CD8^+^ granulocytes. PMNs and PBMCs were mixed together. For fixation of the plasma membrane, the cells were exposed to 4% PFA for 10 min at room temperature. After washing 3 times with PBS, the cells were re-suspended in the autologous serum. The samples were made to react with the PE-labeled anti-CD8α Ab (HIT8a). After removal of unbound Abs, the cells were re-suspended in PBS followed by reaction with the FITC-labeled anti-CD15 Ab (H198), and then served for FCM. For inhibition of actin recruitment, the mixture of PMNs and PBMCs was made to react with CyD (2 µg/ml) for 30 min at 37°C. After the pre-incubation, the cells were made to react with the PE-labeled anti-CD8α Ab (HIT8a). After removal of unbound Abs, the cells were re-suspended in PBS followed by reaction with the FITC-labeled anti-CD15 (H198), and then served for FCM. PE-labeled mouse IgG1 and FITC-labeled mouse IgM were used as isotype-matched controls for HIT8a and H198, respectively. These experiments were repeated 3 times.

Under the mixed culture condition, it is considered that the anti-CD8α Ab binding to CD8 on T cells link granulocytes through the Fc portion of the Ab and cell surface FcγRs on granulocytes. Human resting granulocytes express mainly 2 types of FcγRs, FcγRII (CD32) and FcγRIII (CD16). To examine their contribution to the detection of CD8^+^ granulocytes, blocking assay using the specific Abs to FcγRs was conducted. The representative results are shown in Figure 3A. In the present case, the rate of CD8^+^ granulocytes decreased from 18.5% to 2.20% and from 13.5% to 2.40% by blocking of FcγRII (CD32) and FcγRIII (CD16), respectively. These findings indicated that both FcγRII (CD32) and FcγRIII (CD16) on granulocytes were implicated in the mechanism of detection of CD8^+^ granulocytes.

Next, the requirement of dynamism of plasma membrane and actin recruitment for the CD8 translocation was determined. For this purpose, leukocytes (PMNs+PBMCs) were pretreated with paraformaldehyde (PFA) or cytochalasin D (CyD) in order to fix the plasma membrane or to inhibit the actin recruitment, respectively. After these pre-treatments, the cells were made to react with the PE-labeled anti-CD8α Ab. After removal of unbounded Abs, the cells were re-suspended in PBS followed by reaction with the FITC-labeled anti-CD15 Ab, and then served for FCM. The representative results are shown in Figure 3B. In the present case, the rate of CD8^+^ granulocytes decreased from 9.13% to 0.54% and from 13.6% to 4.06% by the PFA fixation and CyD treatment, respectively.

**Figure 4:**
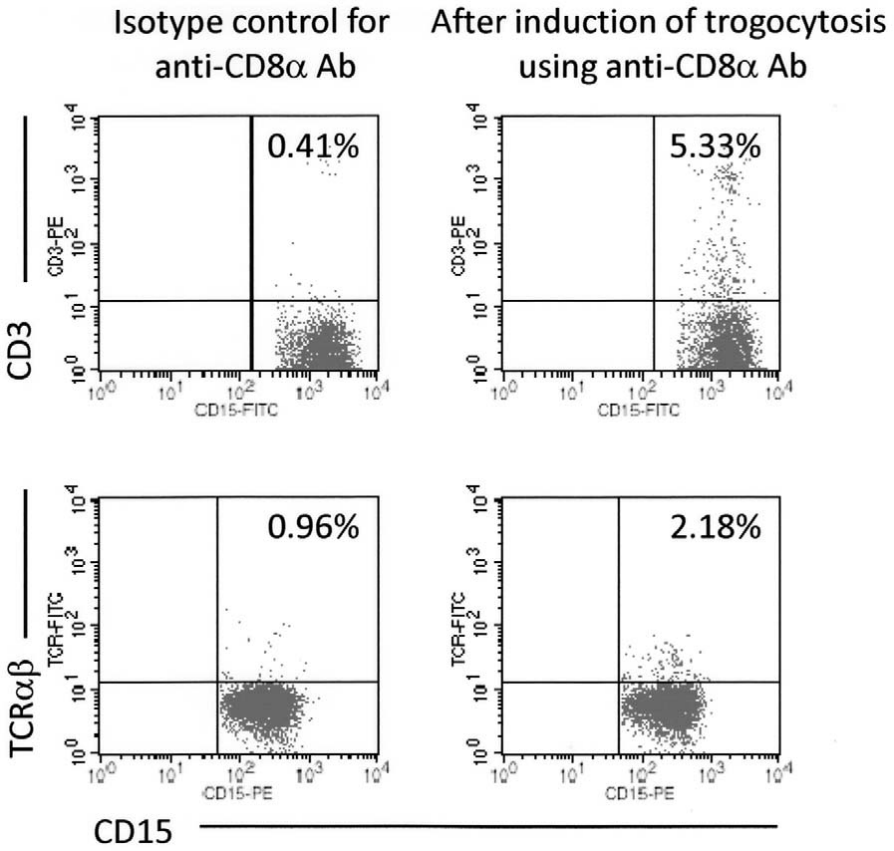
Bystander transfer of CD3 and TCRαβ molecules accompanied by CD8 translocation from T cells to granulocytes. Heparinized blood samples were pre-incubated with the unlabeled anti-CD8α Ab (HIT8a) or mouse IgG1, followed by depletion of erythrocytes, and then re-suspended in PBS. The cells were made to react with the PE-labeled anti-CD3 (UCHT-1) and FITC-labeled anti-CD15 (H198) Abs, or the FITC-labeled anti-TCRαβ (WT31) and PE-labeled anti-CD15 (H198) Abs, followed by serving for FCM. PE or FITC-labeled mouse IgG1 and FITC or PE-labeled mouse IgM were used as isotype-matched controls for HIT8a and H198, respectively. These experiments were repeated 3 times.

In addition, this study determined if CD3 and TCRαβ molecules, which constituted a functional complex with CD8, would be transferred to granulocytes accompanied by CD8 in the CD8 translocation model. The representative results are shown in Figure 4. As a result, CD3 and TCRαβ molecules were transferred from T cells to granulocytes accompanied by CD8, though the degree was lesser than that of CD8. These findings suggested that the plasma membrane fragment, but not a certain molecule alone, was transferred from T cells to granulocytes when these cells were bridged by Abs in the serum.

This CD8 translocation model from T cells to granulocytes is designated as granulocyte FcγR-mediated trogocytosis model.

### Identification of serum factors that contribute to FcγR-mediated trogocytosis

**Figure 5:**
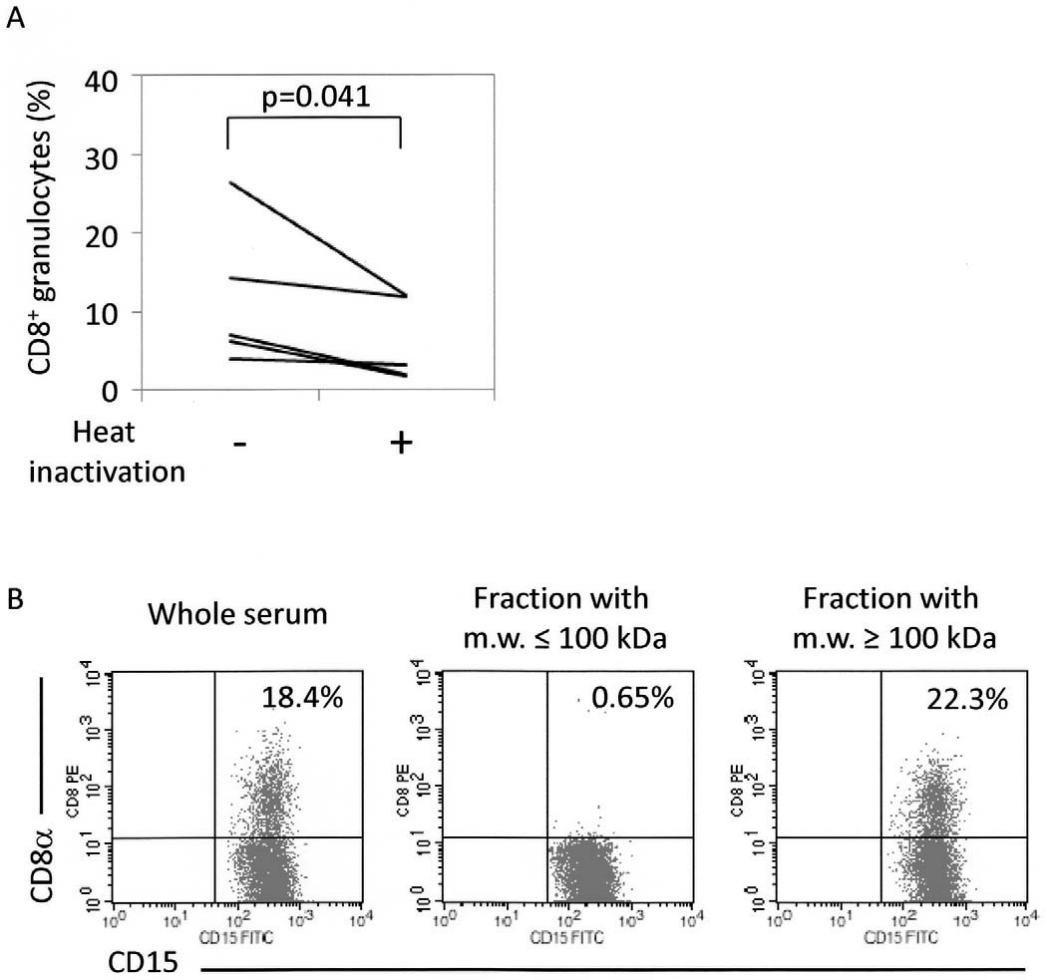
Characterization of serum factors that contribute to FcγR-mediated trogocytosis. (**A**) Heat instability of serum factors that contribute to FcγR-mediated trogocytosis (n = 5). PMNs and PBMCs were mixed in sera, which had been heated at 56°C for 30 min, or sera without heat inactivation. These cells were then made to react with the PE-labeled anti-CD8α Ab (HIT8a). After removal of unbound Abs, the cells were re-suspended in PBS followed by reaction with the FITC-labeled anti-CD15 Ab (H198), and then served for FCM. Student *t*-test for paired samples was applied for statistical analysis. (**B**) Molecular weight range of serum factors that contribute to FcγR-mediated trogocytosis. PMNs and PBMCs were mixed in sera, which had been fractionated into those with molecular weight of more than 100 kDa or less than 100 kDa. These cells were then made to react with the PE-labeled anti-CD8α Ab (HIT8a). After removal of unbound Abs, the cells were re-suspended in PBS followed by reaction with the FITC-labeled anti-CD15 Ab (H198), and then served for FCM. This experiment was repeated 3 times. PE-labeled mouse IgG1 and FITC-labeled mouse IgM were used as isotype-matched controls for HIT8a and H198, respectively.

Our studies have demonstrated the important contribution of serum to FcγR-mediated trogocytosis. In order to investigate the critical factors in the serum, heat stability of the factors was examined. Leukocytes (PMNs+PBMCs) were re-suspended in sera with or without heat inactivation, and then CD8^+^ granulocytes were detected by FCM. As shown in Figure 5A, the rates of CD8^+^ granulocytes were significantly decreased by heat inactivation of the serum (before treatment: 11.6±9.1%; after treatment: 6.2±5.3%). Although the effect of heat inactivation was not necessarily drastic, the rates of CD8^+^ granulocytes were decreased by treatment in all samples examined (n = 5), and there was a statistical significance between before and after the treatment (p = 0.041 in Student's *t*-test for paired samples). Thus, we concluded that the factors that contributed to FcγR-mediated trogocytosis were heat-labile molecules.

Next, the factors were determined whether they belonged to the fraction with molecular weight of less than 100 kDa or more than 100 kDa. Leukocytes (PMNs+PBMCs) were re-suspended in the fractionated serum, and then CD8^+^ granulocytes were detected by FCM. Results clearly indicated that the serum factors were included in the fraction with molecular weight of more than 100 kDa (Figure 5B).

**Figure 6:**
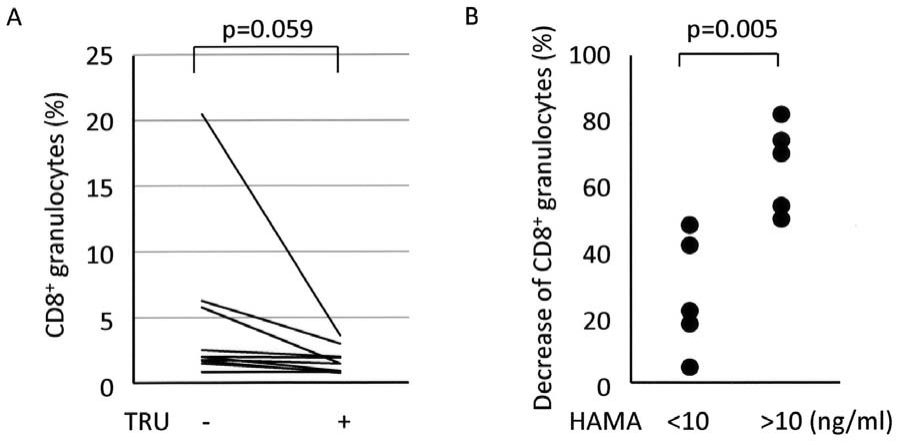
Involvement of HAMA in FcγR-mediated trogocytosis. (**A**) Effects of HAMA blockade on FcγR-mediated trogocytosis (n = 10). Heparinized whole blood samples were added with or without 1 µl of HAMA inhibitor, TRU block, and then made to react with the PE-labeled anti-CD8α (HIT8a) and FITC-labeled anti-CD15 (H198) Abs. After depletion of erythrocytes, these cells were subjected to FCM. PE-labeled mouse IgG1 and FITC-labeled mouse IgM were used as isotype-matched controls for HIT8a and H198, respectively. Student *t*-test for paired samples was applied for statistical analysis. (**B**) Difference in the inhibition rates of FcγR-mediated trogocytosis by the HAMA inhibitor between HAMA^high^ sera and HAMA^low^ sera. The serum samples were divided into 2 groups, including HAMA^high^ serum (more than 10 ng/ml, n = 5) and HAMA^low^ serum (less than 10 ng/ml, n = 5). The decrease rates of CD8^+^ granulocytes by treatment with TRU block were calculated from the data presented in Figure 6A. Student *t*-test for unpaired samples was applied for statistical analysis.

Complements in the classical pathway were initially assumed to be candidates for immune complex-related and heat-labile serum factors with molecular weight of more than 100 kDa that contributed to FcγR-mediated trogocytosis. However, the C1 inhibitor, which effectively blocks the classical pathway of complement activation cascade, did not yield an apparent influence on FcγR-mediated trogocytosis in the CD8 translocation model (data not shown). Next, the possibility that diverse factors cooperated to contribute to FcγR-mediated trogocytosis was considered. In several immunoassays using mouse anti-human Abs, HAMA sometimes interfered in the measurement [5]. Although immunoglobulin itself was not inactivated under the experimental condition (56°C for 30 min) [6], HAMA was speculated as one candidate with molecular weight of more than 100 kDa. In order to evaluate the hypothesis, the effect of HAMA inhibitor, TRU block, on the granulocyte FcγR-mediated trogocytosis-based CD8 translocation model was examined. Interestingly, the inhibitor of HAMA effectively blocked FcγR-mediated trogocytosis though the p-value did not reach a significant level (Figure 6A, n = 10, p = 0.059 in Student's *t*-test for paired samples). Based on this finding, correlation between the rates of CD8^+^ granulocytes and serum concentrations of HAMA was examined. The result revealed no significant correlation between the two (Figure S4). Subsequently, the serum samples were divided into 2 groups, including HAMA^high^ serum (more than 10 ng/ml, n = 5) and HAMA^low^ serum (less than 10 ng/ml, n = 5). The decreased rates of CD8^+^ granulocytes by treatment with TRU block were calculated from the data presented in Figure 6A. As shown in Figure 6B, the decreased rates were significantly higher in HAMA^high^ serum than HAMA^low^ serum (p = 0.005 in Student's *t*-test for unpaired samples).

## Discussion

Exchange of plasma membrane fragments between immune cells that form a conjugate is termed trogocytosis [7]. Trogocytosis was first described on CD8^+^ T cells [8]. CD8^+^ T cells can capture the Ag/class I major histocompatibility complex from Ag-presenting cells via the TCR, when these cells form an immunological synapse. To date, not only CD8^+^ T cells but also other immune cells, including CD4^+^ T cells [9], B cells [10], natural killer cells [11], dendritic cells [12], monocytes [13], and macrophages [14], are confirmed to perform trogocytosis. This mechanism is called adhesion molecule-mediated trogocytosis. Recently, another mechanism of trogocytosis mediated by Ag/Ab immune complexes and FcγRs, so to say FcγR-mediated trogocytosis, is advocated [4]. The best known cells that can perform FcγR-mediated trogocytosis are monocytes. Contrary to the adhesion molecule-mediated trogocytosis, it remains elusive as to what kind of cells with FcγR expression other than monocytes can perform trogocytosis in the FcγR-dependent manner. In the present study, we focused on granulocytes, the largest population with FcγR expression in peripheral blood, and demonstrated that granulocytes could perform FcγR-mediated trogocytosis similar to monocytes.

Briefly, CD8^+^ granulocytes were detected in human peripheral blood samples by the following protocol: 1) Heparinized whole blood samples were made to react with the PE-labeled anti-CD8 Ab, 2) After removal of erythrocytes, the cells were re-suspended in PBS, 3) The cells were allowed to react with the FITC-labeled anti-CD15 Ab, and 4) FCM was performed. Our preliminary results showed that CD8^+^ granulocytes were detected more clearly with the use of this protocol than the conventional whole blood erythrocyte lysis method (data not shown). Although further investigations are needed to clarify the mechanism, the binding potential of FcγRs was possibly increased in the steps after the first wash. At any rate, since granulocytes did not produce CD8 mRNAs and CD8 molecules were not detected on purified granulocytes, the results were regarded as false-positive. Analysis of the mechanism revealed that CD8 molecules on T cells were transferred to granulocytes when whole blood samples were incubated with the anti-CD8 Ab. For completion of the CD8 translocation from T cells to granulocytes, cell-to-cell contact between T cells and granulocytes as well as binding of the Fc portion of the Ab and FcγRs on granulocytes, were required. In addition, CD3 and TCR molecules that constituted a functional complex with CD8 were transferred from T cells to granulocytes accompanied by the translocation of CD8. This bystander transfer is a feature of FcγR-mediated trogocytosis which means that the fragment of plasma membrane but not a certain molecule alone is transferred via bridging Abs [2], [4]. The lower degree of the bystander transfer compared to the CD8 translocation could be explained by intermolecular distance or weak association strength. These findings also corresponded to the previous report that demonstrated the mutual exchange of plasma membrane between granulocytes and tumor cells mediated by tumor-targeting Abs [15].

The detection rates of CD8^+^ granulocytes were well correlated to those of CD8^+^ monocytes, and individual differences in the capacity of FcγR-mediated trogocytosis were present among samples. Our studies have demonstrated that depletion of serum retarded the CD8 detection on monocytes and granulocytes; thus, these findings indicated the essential contribution of serum factors to FcγR-mediated trogocytosis. Furthermore, the amount or activity of the putative factors was suggested to regulate the occurrence of FcγR-mediated trogocytosis. Based on the concept that the identification of such factors should be important for the quality control of FCM, the serum factors were searched for using the granulocyte FcγR-mediated trogocytosis model. Ultimately, HAMA was suggested as one factor that could contribute to FcγR-mediated trogocytosis.

In brief, investigation with granulocyte FcγR-mediated trogocytosis model determined the serum factors that contributed to FcγR-mediated trogocytosis as heat-labile molecules with molecular weight of more than 100 kDa. Complements in the classical pathway were initially assumed as candidates; however, this possibility was not likely because the C1 inhibitor did not yield an obvious influence on FcγR-mediated trogocytosis. Therefore, based on the consideration that diverse factors cooperated to contribute to FcγR-mediated trogocytosis, HAMA was included as candidate with molecular weight of more than 100 kDa but without heat-instability. Interestingly, the HAMA inhibitor, TRU block, inhibited FcγR-mediated trogocytosis effectively. Moreover, the inhibition rates of CD8^+^ granulocytes by the treatment with TRU block were significantly higher in HAMA^high^ serum than HAMA^low^ serum. Although the serum concentrations of HAMA were not directly correlated to the rates of CD8^+^ granulocytes, this does not necessarily deny the contribution of HAMA because HAMA could possibly function in synergy with undetermined factors to promote FcγR-mediated trogocytosis.

HAMA can interfere with immunoassay measurements that utilize mouse anti-human Abs [5]. There are several possible mechanisms for assay interference. For example, in sandwich immunoassays, HAMA can either bridge the captured and diagnostic Abs then produce false-positive results or block the binding of the diagnostic Abs then yield false-negative results. Therefore, understanding of the assay interference mechanism of HAMA is very critical for quality control of immunoassay measurements. The present study indicated a possible mechanism for assay interference of HAMA via promotion of FcγR-mediated trogocytosis.

In the present study, we focused on the false-positive results in whole blood erythrocyte lysis method in FCM. In this protocol, FcγR blockers are unnecessary because considerable amount of human IgG, which can mask FcγRs, is included in the sample itself. However, since FcγR-mediated trogocytosis was not induced by Abs without Fc portion and was inhibited by blocking Abs to FcγRs, strong inhibition of the FcγR is still desirable. Although the precise mechanism of FcγR-mediated trogocytosis has not been revealed, the HAMA inhibitor, TRU block, inhibited FcγR-mediated trogocytosis effectively. Therefore, the usage of HAMA blockers may be a reasonable choice to prevent false-positive results. Alternatively, the usage of buffers containing membrane fixative reagents should lead to definite results because the fixation of membrane also inhibited FcγR-mediated trogocytosis effectively. Nevertheless, further efforts to identify the serum factors that contribute to FcγR-mediated trogocytosis in synergy with heterophilic Abs are needed in order to achieve a high quality measurement of FCM.

Lastly, another significance of this study is in relation to malignant and autoimmune diseases. FcγR-mediated trogocytosis is well known as the phenomenon wherein CD20 molecules on malignant B cells are lost after infusion of the humanized anti-CD20 monoclonal Abs, rituximab [16], [17]. In other words, FcγR-mediated trogocytosis can occur not only *in vitro* but also *in vivo*. Furthermore, it can be speculated that FcγR-mediated trogocytosis plays a certain role in patients with production of autoantibodies in serum. Therefore, investigation into the underlying mechanism of FcγR-mediated trogocytosis using *in vitro* model system is important to reveal the biologically substantial significance of FcγR-mediated trogocytosis *in vivo*.

## Materials and Methods

### Human peripheral blood samples

This study was conducted with permission of the Institutional Clinical Research Committee in Hokkaido University Hospital (No. 0903-0398). Human peripheral blood samples were obtained from healthy volunteers with written informed consent. No one had any medical history of autoimmune disease, recent infection, or neoplasm.

### Antibodies (Abs)

Mouse anti-human monoclonal Abs used were anti-CD3 (UCHT-1, BD Pharmingen, Franklin Lakes, NJ), anti-CD8α (HIT8a and RPA-T8, BD Pharmingen), anti-CD8β (2ST8.5H7, Serotec, Oxford, UK), anti-CD14 (M5E2, BD Pharmingen), anti-CD15 (H198, BD Pharmingen), anti-CD16 (3G8, BD Pharmingen), anti-CD32 (AT10, Abcam, Cambridge, MA), and anti-TCRαβ (WT31, BD Pharmingen) Abs. IgG1 was the isotype, except for 2ST8.5H7, M5E2 and H198. The isotype of 2ST8.5H7 and M5E2 was IgG2a and that of H198 was IgM. Isotype-matched mouse IgG1 (MOPC-21, BD Pharmingen), IgG2a (DAK-GO5, Dako), and IgM (MM-30, BioLegend, San Diego, CA) served as controls.

### Fluorescence-labeling of CD8α^+^ monocytes and CD8α^+^ granulocytes in human peripheral blood samples

Heparinized human peripheral blood samples were utilized for direct immunostaining without removal of plasma. CD8α^+^CD14^+^ cells (CD8^+^ monocytes) were detected as described previously [2]. For detection of CD8α^+^ cells in granulocytes (CD15^+^ PMNs), blood samples (100 µl) were made to react with 0.1 µg of the PE-labeled anti-CD8α Ab for 20 min at room temperature. After depletion of erythrocytes by treatment with ammonium chloride as described previously [2], the cells were re-suspended in 100 µl of PBS, and then allowed to react with 0.1 µg of the FITC-labeled anti-CD15 Ab for 20 min at room temperature. PE-labeled mouse IgG1 and FITC-labeled mouse IgM were used as isotype-matched controls for the anti-CD8α and anti-CD15 Abs, respectively.

### Flow cytometry (FCM)

The fluorescence-labeled cells were assayed using FACS Calibur (BD Biosciences, Franklin Lakes, NJ) with CellQuest software (BD Biosciences). Unlabeled cells were used to set-up the machine. For multiple staining, single fluorescence-labeled cells were used for compensation of fluorescence levels. CD8α^+^CD15^+^ PMNs were designated as CD8^+^ granulocytes.

### Separation of cells

PMNs were separated from heparinized human peripheral blood using Polymorphprep (Axis-Shield, Norton, MA) according to the manufacturer's instruction. For separation of granulocytes, PMNs were re-suspended in PBS, and then made to react with the FITC-labeled anti-CD15 Ab for 20 min at room temperature. After removal of unbound Abs, the cells were re-suspended in MACS buffer (0.5% BSA and 2 mM EDTA in PBS), made to react with anti-FITC MicroBeads (Miltenyi Biotec) for 10 min at 4°C, and then CD15^+^ cells were positively selected using the MACS system (Miltenyi Biotech) as described previously [2]. PBMCs were separated from heparinized peripheral blood using Ficoll-Paque Plus (GE-Healthcare, Tokyo, Japan). For separation of T cells, PBMCs were re-suspended in MACS buffer, made to react with CD3 MicroBeads (Miltenyi Biotech) for 15 min at 4°C, and then CD3^+^ cells were positively selected using the MACS system. FCM revealed that the purity of granulocytes (CD15^+^ PMNs) and T cells (CD3^+^ cells) separated using the MACS system was more than 95%.

### RT-PCR

Heparinized peripheral blood sample (5 ml) was incubated with 5 µg of the PE-labeled anti-CD8α Ab for 20 min at room temperature. Subsequently, granulocytes were separated. FCM revealed that CD8α^+^ cells were present in the purified granulocytes. For the control study, CD3^+^ cells, which contained CD8^+^ T cells, were also separated from heparinized peripheral blood. Total RNAs were extracted from the purified granulocytes (CD15^+^ PMNs) and T cells (CD3^+^ cells) using RNeasy Mini kit (Qiagen, Alameda, CA) according to manufacturer's instructions. Samples were treated with DNase I for 15 min at room temperature to avoid contamination of DNA. The purified RNAs were reverse-transcribed by Superscript III reverse transcriptase (Invitrogen, Carlsbag, CA) using oligo-dT primers as described previously [2]. The cDNA concentration was estimated by absorbance, and 100 ng of cDNA served as templates. PCR was performed in 25 µl reaction with specific primer pairs (25 µM) listed below; glyceraldehyde-3-phosphate dehydrogenase (GAPDH): 5′-AGCGAGATCCCTCCAAAATC-3′ (sense) and 5′-GGCAGAGATGATGACCCTTT-3′ (antisense), CD3ε: 5′-GTGGACATCTGCATCACTGG-3′ (sense) and 5′-TAGTCTGGGTTGGGAACAGG-3′ (antisense), CD8α: 5′-GCGAGACAGTGGAGCTGAA-3′ (sense) and 5′-GGCTTGTTTTGGGAGAGGTA-3′ (antisense), CD8β: 5′-GGCTGTGGCTCCTCTTGG-3′ (sense) and 5′-ATTTTAGCCTCGCAGGACAG-3′ (antisense), and CD11b: 5′-CAGCATAAACCACCCCATCT-3′ (sense) and 5′-GGTTCTGGGCATGTTGTTCT-3′ (antisense). PCR amplification was performed as 35 cycles of 30 sec at 95°C, 30 sec at 56°C, and 30 sec at 72°C. After the final cycle, the PCR products were incubated at 72°C for 7 min to complete polymerization. The PCR products were run on 2% agarose gel.

### Serum removal assay

PMNs (0.5×10^6^) and PBMCs (0.5×10^6^) separated from heparinized peripheral blood were mixed in 100 µl of PBS or autologous serum. These cells were incubated with 0.1 µg of the PE-labeled anti-CD8α Ab for 20 min at room temperature. After removal of unbound Abs, the cells were re-suspended in 100 µl of PBS followed by reaction with 0.1 µg of the FITC-labeled anti-CD15 Ab for 20 min at room temperature, and then served for FCM. PE-labeled mouse IgG1 and FITC-labeled mouse IgM were used as isotype-matched controls for the anti-CD8α and anti-CD15 Abs, respectively.

### Co-culture assay

PMNs and T cells were separated from heparinized blood samples. PMNs (0.5×10^6^) were cultured with or without T cells (0.5×10^6^) in the autologous serum in 24-well plates for 30 min at 37°C. In the co-culture wells, PMNs were cultured separately from T cells using 0.4 µm pore-sized transwell chambers (BD Falcon, Franklin Lakes, NJ) or mixed together with T cells. Subsequently, the cells were made to react with 0.1 µg of the PE-labeled anti-CD8α Ab for 20 min at room temperature. After removal of unbound Abs, the cells were re-suspended in 100 µl of PBS followed by reaction with 0.1 µg of the FITC-labeled anti-CD15 Ab for 20 min at room temperature, and then served for FCM. PE-labeled mouse IgG1 and FITC-labeled mouse IgM were used as isotype-matched controls for the anti-CD8α and anti-CD15 Abs, respectively.

### FcγR blocking assay

Heparinized blood samples (100 µl) were pre-incubated with 3 µg of the anti- FcγRII (CD32) Ab for 30 min or with 2 µg of the FITC-labeled anti-FcγRIII (CD16) Ab for 45 min at room temperature according to the previous literatures [2], [18]. Unlabeled or FITC-labeled mouse IgG1 was used as an isotype-matched control. The FITC-labeled anti-FcγRIII (CD16) Abs were used reluctantly because the unlabeled Abs were not available. After the pre-incubation, 0.1 µg of the PE or PECy5-labeled anti-CD8α Ab was added, and the samples were further incubated for 20 min at room temperature. After depletion of erythrocytes, the cells were re-suspended in 100 µl of PBS followed by reaction with 0.1 µg of the FITC or PE-labeled anti-CD15 Ab for 20 min at room temperature, and then served for FCM. PE or PECy5-labeled mouse IgG1 and FITC or PE-labeled mouse IgM were used as isotype-matched controls for the anti-CD8α and anti-CD15 Abs, respectively.

### Effect of plasma membrane fixation and inhibition of actin recruitment

PMNs (0.5×10^6^) and PBMCs (0.5×10^6^) were mixed together. For fixation of the plasma membrane, the cells were exposed to 4% paraformaldehyde (PFA) for 10 min at room temperature. After washing 3 times with PBS, the cells were re-suspended in 100 µl of the autologous serum. The samples were made to react with 0.1 µg of the PE-labeled anti-CD8α Ab for 20 min at room temperature. After removal of unbound Abs, the cells were re-suspended in 100 µl of PBS followed by reaction with 0.1 µg of the FITC-labeled anti-CD15 Ab for 20 min at room temperature, and then served for FCM. For inhibition of actin recruitment, the mixture of PMNs (0.5×10^6^) and PBMCs (0.5×10^6^) was made to react with 2 µg/ml cytochalasin D (CyD, Sigma-Aldrich) for 30 min at 37°C. After the pre-incubation, 0.1 µg of the PE-labeled anti-CD8α Ab was added, and the samples were further incubated for 20 min at room temperature. After removal of unbound Abs, the cells were re-suspended in 100 µl of PBS followed by reaction with 0.1 µg of the FITC-labeled anti-CD15 Ab for 20 min at room temperature, and then served for FCM. PE-labeled mouse IgG1 and FITC-labeled mouse IgM were used as isotype-matched controls for the anti-CD8α and anti-CD15 Abs, respectively.

### Detection of bystander transfer

Heparinized blood samples (100 µl) were pre-incubated with 0.1 µg of the unlabeled anti-CD8α Ab or mouse IgG1 for 20 min at room temperature, followed by depletion of erythrocytes, and then re-suspended in PBS (100 µl). The cells were made to react with 0.1 µg of the PE-labeled anti-CD3 and FITC-labeled anti-CD15 Abs, or the FITC-labeled anti-TCRαβ and PE-labeled anti-CD15 Abs for 20 min at room temperature, followed by serving for FCM. PE or FITC-labeled mouse IgG1 and FITC or PE-labeled mouse IgM were used as isotype-matched controls for the anti-CD8α and anti-CD15 Abs, respectively.

### Characteristics of serum factors that contribute to FcγR-mediated trogocytosis

First, PMNs (0.5×10^6^) and PBMCs (0.5×10^6^) were mixed in 100 µl of sera, which had been heated at 56°C for 30 min, or sera without heat inactivation. Next, PMNs (0.5×10^6^) and PBMCs (0.5×10^6^) were mixed in 100 µl of sera, which had been fractionated into those with molecular weight of more than 100 kDa or less than 100 kDa using Amicon Ultra-4 100K device (Millipore, Jaffrey, NH). These cells were then made to react with 0.1 µg of the PE-labeled anti-CD8α Ab for 20 min at room temperature. After removal of unbound Abs, the cells were re-suspended in 100 µl of PBS followed by reaction with 0.1 µg of the FITC-labeled anti-CD15 Ab for 20 min at room temperature, and then served for FCM. PE-labeled mouse IgG1 and FITC-labeled mouse IgM were used as isotype-matched controls for the anti-CD8α and anti-CD15 Abs, respectively.

### Effects of complement inhibition and blockade of HAMA on FcγR-mediated trogocytosis

First, PMNs (0.5×10^6^) and PBMCs (0.5×10^6^) were re-suspended in the autologous serum (100 µl) with or without 200 µg/ml of C1 inhibitor (Merck Millipore, Schwalbach, Germany). These cells were then made to react with 0.1 µg of the PE-labeled anti-CD8αAb for 20 min at room temperature. After removal of unbound Abs, the cells were re-suspended in 100 µl of PBS followed by reaction with 0.1 µg of the FITC-labeled anti-CD15 Ab for 20 min at room temperature, and then served for FCM. Next, whole blood samples were added with or without 1 µl of HAMA inhibitor, TRU block (Meridian Life Science, Saco, ME), and then made to react with 0.1 µg of the PE-labeled anti-CD8α and FITC-labeled anti-CD15 Abs for 20 min at room temperature. After depletion of erythrocytes, these cells were subjected to FCM. PE-labeled mouse IgG1 and FITC-labeled mouse IgM were used as isotype-matched controls for the anti-CD8α and anti-CD15 Abs, respectively.

### Quantification of HAMA in serum

HAMA in the serum was quantified using HAMA ELISA kit (Abnova, Heidelberg, Germany) according to manufacturer's instruction.

## Supporting Information

Figure S1 **Detection of CD8^+^ granulocytes using other anti-CD8α and anti-CD8β Abs.** Heparinized whole blood samples were made to react with the FITC-labeled anti-CD8α (RPA-T8) (**A**) or Alexa 488-labeled anti-CD8β (2ST8.5H7) (**B**) Abs. After depletion of erythrocytes, the cells were re-suspended in PBS, and then allowed to react with the PE-labeled anti-CD15 Ab (H198). FITC-labeled mouse IgG1, Alexa 488-labeled mouse IgG2a, and PE-labeled mouse IgM were used as isotype-matched controls for RPA-T8, 2ST8.5H7, and H198, respectively. The CD15^+^ PMNs (granulocytes) were examined for the expression of CD8α and CD8β.
(TIF)

Figure S2 **Presence of CD8^+^ cells in the granulocyte sample.** This data corresponded to Figure 2A.
(TIF)

Figure S3 **FSC/SSC profiles of the co-culture experiments of PMNs and T cells.** These data corresponded to Figure 2C. The R1 gate in the left panel represented the characteristic profile of single-cell PMNs. In the mixed culture of PMNs and T cells (right panel), the cells with the identical FSC/SSC profile of single-cell PMNs (within the R1 gate) were included in the assay.
(TIF)

Figure S4 **Correlation between the rates of CD8^+^ granulocytes and serum concentrations of HAMA.** Statistical analysis revealed no significant correlation between the two.
(TIF)

## References

[1] Renzi P, Ginns LC (1987) Analysis of T cell subsets in normal adults. Comparison of whole blood lysis technique to Ficoll-Hypaque separation by flow cytometry. J Immunol Methods 98: 53–56. 2951443 10.1016/0022-1759(87)90434-0

[2] Iwasaki S, Masuda S, Baba T, Tomaru U, Katsumata K, et al (2011) Plasma-dependent, antibody- and Fcγ receptor-mediated translocation of CD8 molecules from T cells to monocytes. Cytometry A 79: 46–56. 21182182 10.1002/cyto.a.20984

[3] Joly E, Hudrisier D (2003) What is trogocytosis and what is its purpose? Nat Immunol 4: 815. 12942076 10.1038/ni0903-815

[4] Daubeuf S, Lindorfer MA, Taylor RP, Joly E, Hudrisier D (2010) The direction of plasma membrane exchange between lymphocytes and accessory cells by trogocytosis is influenced by the nature of the accessory cell. J Immunol 184: 1897–1908. 20089699 10.4049/jimmunol.0901570

[5] Klee GG (2000) Human anti-mouse antibodies. Arch Pathol Lab Med 124: 921–923. 10835540 10.5858/2000-124-0921-HAMA

[6] Ishikawa T, Ito T, Endo R, Nakagawa K, Sawa E, et al (2010) Influence of pH on heat-induced aggregation and degradation of therapeutic monoclonal antibodies. Biol Pharm Bull 33: 1413–1417. 20686240 10.1248/bpb.33.1413

[7] Ahmed KA, Xiang J (2011) Mechanisms of cellular communication through intercellular protein transfer. J Cell Mol Med 15: 1458–1473. 20070437 10.1111/j.1582-4934.2010.01008.x PMC3823191

[8] Arnold PY, Davidian DK, Mannie MD (1997) Antigen presentation by T cells: T cell receptor ligation promotes antigen acquisition from professional antigen-presenting cells. Eur J Immunol 27: 3198–3205. 9464806 10.1002/eji.1830271217

[9] Puaux AL, Campanaud J, Salles A, Preville X, Timmerman B, et al (2006) A very rapid and simple assay based on trogocytosis to detect and measure specific T and B cell reactivity by flow cytometry. Eur J Immunol 36: 779–788. 16482513 10.1002/eji.200535407

[10] Batista FD, Iber D, Neuberger MS (2001) B cells acquire antigen from target cells after synapse formation. Nature 411: 489–494. 11373683 10.1038/35078099

[11] Carlin LM, Eleme K, McCann FE, Davis DM (2001) Intercellular transfer and supramolecular organization of human leukocyte antigen C at inhibitory natural killer cell immune synapses. J Exp Med 194: 1507–1517. 11714757 10.1084/jem.194.10.1507 PMC2193674

[12] Herrera OB, Golshayan D, Tibbott R, Salcido Ochoa F, James MJ, et al (2004) A novel pathway of alloantigen presentation by dendritic cells. J Immunol 173: 4828–4837. 15470023 10.4049/jimmunol.173.8.4828

[13] Xu H, Dhanireddy KK, Kirk AD (2006) Human monocytes as intermediaries between allogeneic endothelial cells and allospecific T cells: a role for direct scavenger receptor-mediated endothelial membrane uptake in the initiation of alloimmunity. J Immunol 176: 750–761. 16393958 10.4049/jimmunol.176.2.750

[14] Harvey BP, Quan TE, Rudenga BJ, Roman RM, Craft J, et al (2008) Editing antigen presentation: antigen transfer between human B lymphocytes and macrophages mediated by class A scavenger receptors. J Immunol 181: 4043–4051. 18768860 10.4049/jimmunol.181.6.4043 PMC2701691

[15] Horner H, Frank C, Dechant C, Repp R, Glennie M, et al (2007) Intimate cell conjugate formation and exchange of membrane lipids precede apoptosis induction in target cells during antibody-dependent, granulocyte-mediated cytotoxicity. J Immunol 179: 337–345. 17579054 10.4049/jimmunol.179.1.337

[16] Beum PV, Kennedy AD, Williams ME, Lindorfer MA, Taylor RP (2006) The shaving reaction: rituximab/CD20 complexes are removed from mantle cell lymphoma and chronic lymphocytic leukemia cells by THP-1 monocytes. J Immunol 176: 2600–2609. 16456022 10.4049/jimmunol.176.4.2600

[17] Li Y, Williams ME, Cousar JB, Pawluczkowycz AW, Lindorfer MA, et al (2007) Rituximab-CD20 complexes are shaved from Z138 mantle cell lymphoma cells in intravenous and subcutaneous SCID mouse models. J Immunol 179: 4263–4271. 17785867 10.4049/jimmunol.179.6.4263

[18] Dhodapkar KM, Kaufman JL, Ehlers M, Banerjee DK, Bonvini E, et al (2005) Selective blockade of inhibitory Fcγ receptor enables human dendritic cell maturation with IL-12p70 production and immunity to antibody-coated tumor cells. Proc Natl Acad Sci USA 102: 2910–2915. 15703291 10.1073/pnas.0500014102 PMC549508

