# Supplementary figures and images for: Mechanism of Fcγ Receptor-Mediated Trogocytosis-Based False-Positive Results in Flow Cytometry
Source: PLoS One. 2012 Dec 27;7(12):e52918. doi: 10.1371/journal.pone.0052918 (PMC3531343; doi:10.1371/journal.pone.0052918)

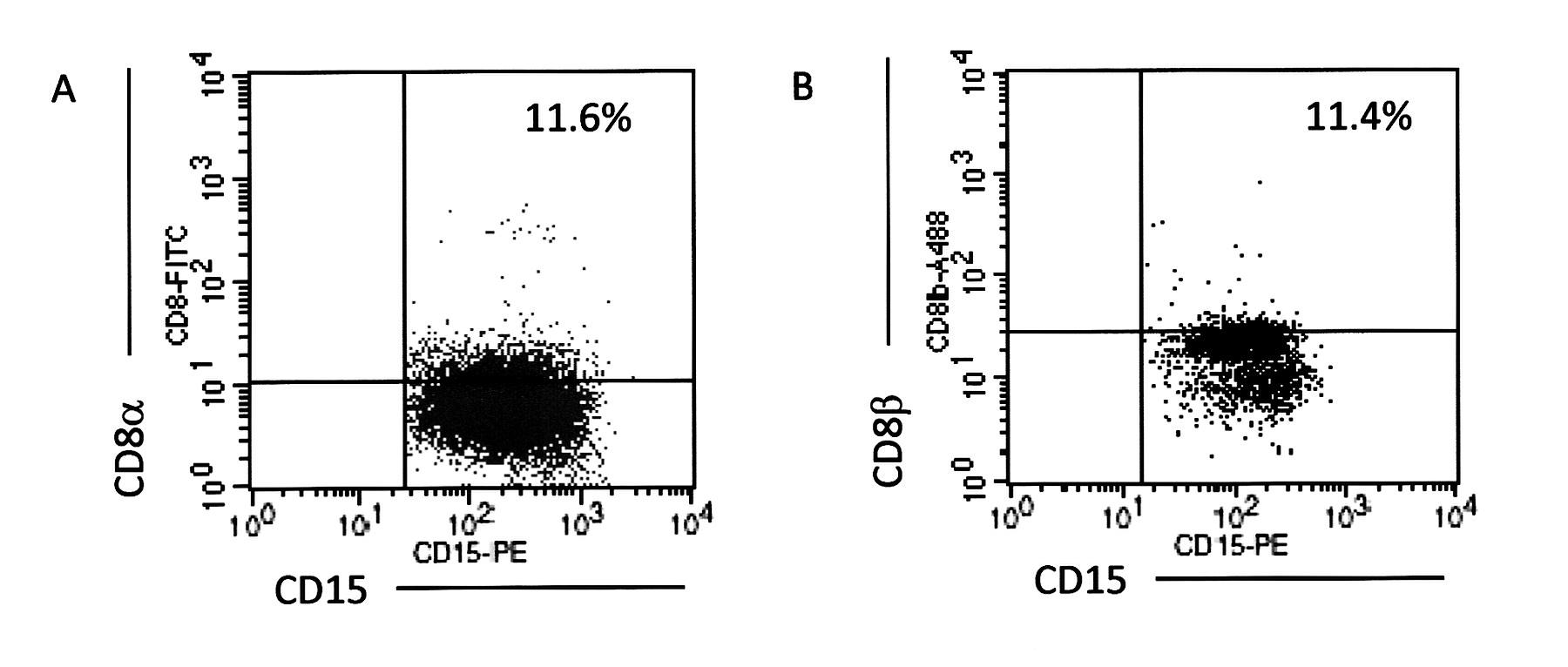

Supplement: Figure S1 — Detection of CD8+ granulocytes using other anti-CD8α and anti-CD8β Abs. Heparinized whole blood samples were made to react with the FITC-labeled anti-CD8α (RPA-T8) (A) or Alexa 488-labeled anti-CD8β (2ST8.5H7) (B) Abs. After depletion of erythrocytes, the cells were re-suspended in PBS, and then allowed to react with the PE-labeled anti-CD15 Ab (H198). FITC-labeled mouse IgG1, Alexa 488-labeled mouse IgG2a, and PE-labeled mouse IgM were used as isotype-matched controls for RPA-T8, 2ST8.5H7, and H198, respectively. The CD15+ PMNs (granulocytes) were examined for the expression of CD8α and CD8β. (TIF) [file pone.0052918.s001.tif]

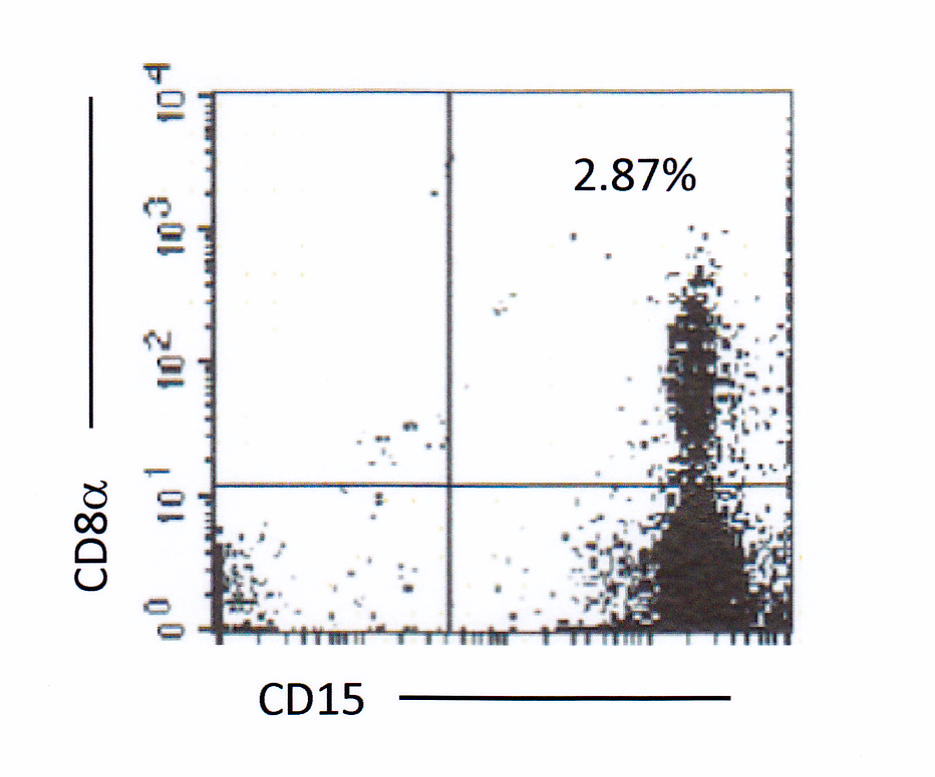

Supplement: Figure S2 — Presence of CD8+ cells in the granulocyte sample. This data corresponded to Figure 2A. (TIF) [file pone.0052918.s002.tif]

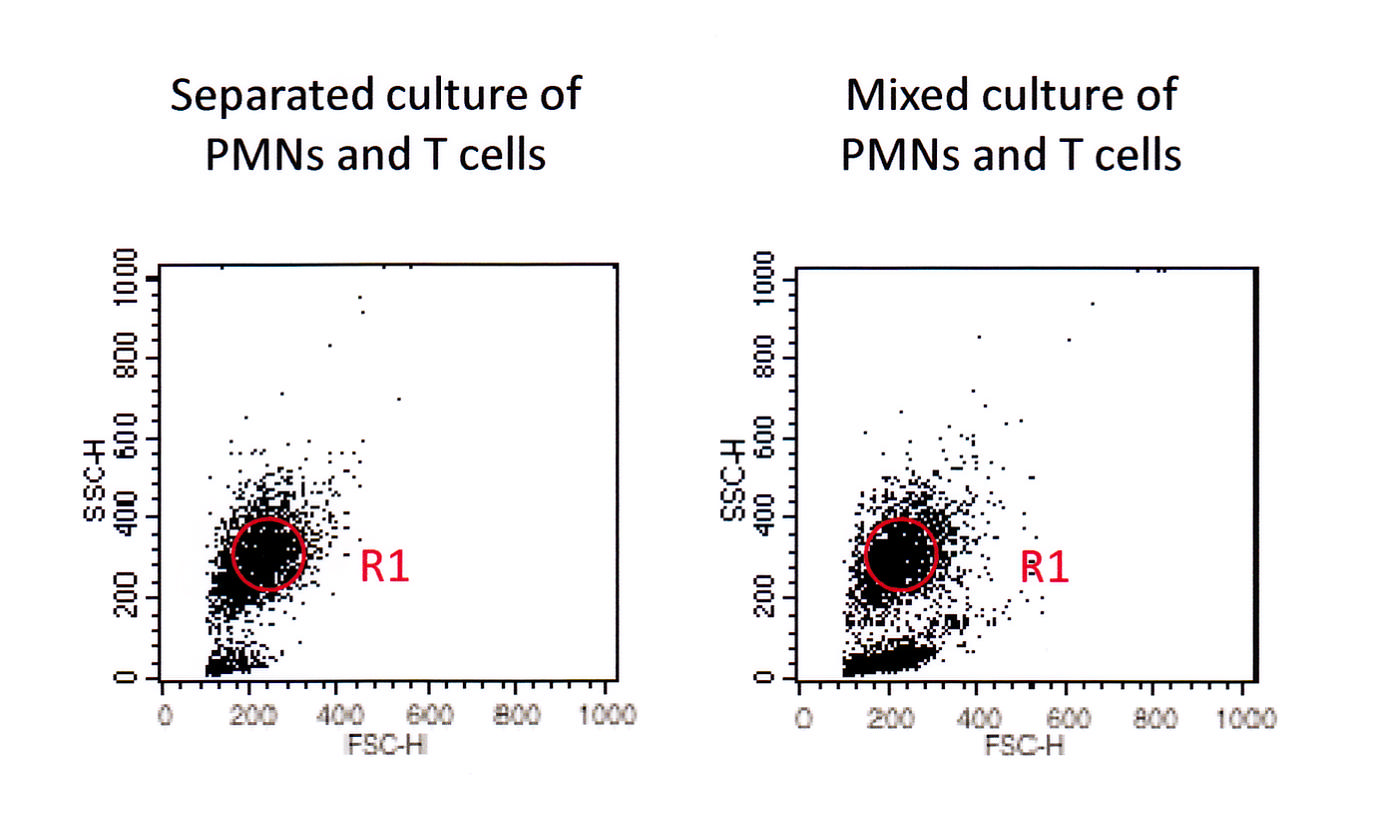

Supplement: Figure S3 — FSC/SSC profiles of the co-culture experiments of PMNs and T cells. These data corresponded to Figure 2C. The R1 gate in the left panel represented the characteristic profile of single-cell PMNs. In the mixed culture of PMNs and T cells (right panel), the cells with the identical FSC/SSC profile of single-cell PMNs (within the R1 gate) were included in the assay. (TIF) [file pone.0052918.s003.tif]

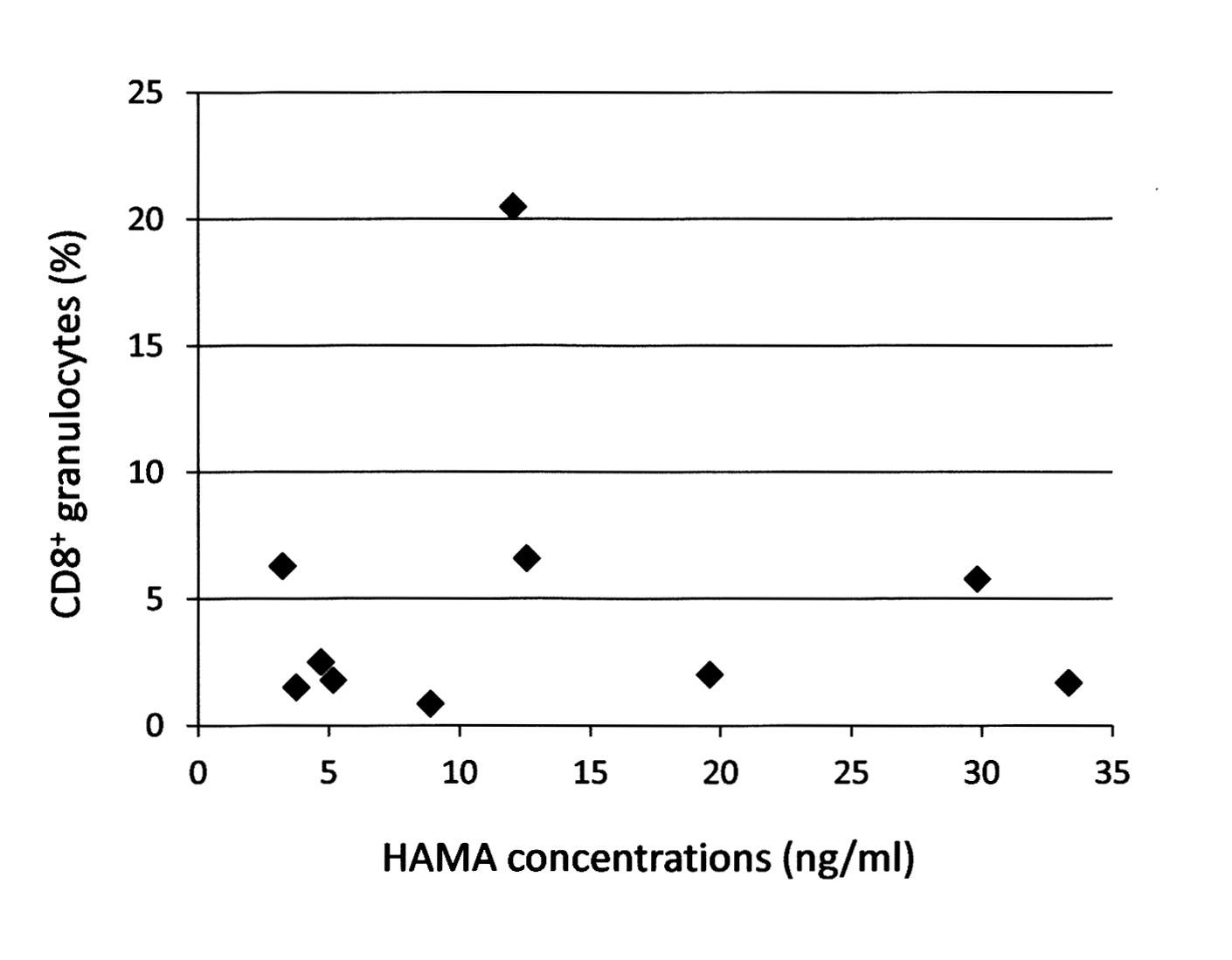

Supplement: Figure S4 — Correlation between the rates of CD8+ granulocytes and serum concentrations of HAMA. Statistical analysis revealed no significant correlation between the two. (TIF) [file pone.0052918.s004.tif]
